
JOURNAL INFORMATION
==============================
NLM Title Abbreviation: Ann R Coll Surg Engl
Journal ID: Ann R Coll Surg Engl
NLM Journal ID: 7506860
Journal ID: ann

Annals of The Royal College of Surgeons of England

ISSN: 0035-8843
EISSN: 1478-7083
Publisher: Royal College of Surgeons of England

ARTICLE INFORMATION
==============================
PMCID: PMC11847617
DOI: 10.1308/rcsann.2025.0010
Article ID: rcsann.2025.0010
Article version: 1
Subjects: ASiT Innovation Summit Oral Presentations

ASiT Innovation Summit Oral Presentations

Print publication date: 2025 Feb 24
Volume: 107
Issue: Suppl 1
First page: S1
Last page: S14
Copyright: Copyright © 2025, The Authors
Copyright year: 2025
License: This is an open-access article distributed under the terms of the Creative Commons Attribution 4.0 International License, which permits unrestricted use, distribution, reproduction, and adaptation in any medium, provided the original work is properly attributed.
License URL: https://creativecommons.org/licenses/by/4.0/

==============================

## Artificial intelligence in surgery

**Aim:** Artificial intelligence (AI) is driving innovative solutions in healthcare, potentially leading to improved outcomes. Despite its widespread use and numerous benefits in women's health within high-income countries, its application remains disproportionately limited in low- and middle-income countries (LMICs). This paper investigates the factors contributing to this inequality and proposes strategies to address them.
**Method:** We conducted narrative review using published literature focused on emerging opportunities and barriers to implementation of AI in healthcare systems in LMICs and its interconnection with maternal health.
**Results:** Women face unique health challenges that are further compounded by the discrimination and violence they experience due to their gender. AI-powered women's health solutions in LMICs are largely non-existent majorly due to lack of technical infrastructure, dearth of structured data, socioeconomic barriers, and poor health regulations. Furthermore, populations in LMICs may face increased inequalities from the use of AI, due to the high cost of medical care and a lack of the technical expertise needed for effective implementation. More funding is required to incorporate AI into the existing maternal healthcare. Designing sustainable health policies and a robust legal framework will help reduce inequalities and disparities in access faced by LMICs.
**Conclusions:** This review highlights AI's potential to enhance women's health. However, addressing the barriers to its adoption is crucial for reducing inequalities in LMICs and ultimately improving access to quality maternal healthcare.

**Aim:** Evaluating pancreatic steatosis during organ donation plays an important role in utilisation decisions, but current methods rely on subjective visual assessments. Unlike other organs, the pancreas cannot be biopsied if intended for transplantation. Artificial intelligence (AI) models offer an objective alternative, but only if computers can accurately identify the organ from surrounding tissue (image segmentation). We present the first automated segmentation model designed for pancreas photographs.
**Method:** Two independent anonymized image datasets were used of pancreata on the backbench. The first dataset, comprising 264 images, was split 75:25 for training and testing. The second dataset contained 58 images for external validation. Ground truth annotations were verified by a transplant surgeon. We trained Detectron2 and YOLOv8, two open-source models, for automated segmentation.
**Results:** Compared to human segmentation, in internal validation Detectron2 and YOLOv8 achieved accuracies of 98%, Dice coefficients (DIC) of 95% and 96%, and Areas Under the Receiver Operating Characteristic Curves (AUC) of 96% and 97%, respectively. In external validation, Detectron2 and YOLOv8 recorded accuracies of 97%, DIC of 93% and 94%, and AUC of 96% and 97%, respectively. Human annotation took a median of 64 (IQR:54-78) seconds. Detectron2 was 64 times faster at 0.95 (0.87-1.07) seconds, while YOLOv8 was 640 times faster at 0.10 (0.09-0.10) seconds.
**Conclusions:** Automated annotation enables significantly faster pancreas segmentation compared to manual methods. Future work will aim to refine models to distinguish between different pancreatic regions and ensure AI assessments are not confounded by nearby visceral fat.

**Aim:** Morphometric features have been shown to influence knee OA progression. Phenotyping of patients facilitates more personalised management and understanding. This study aims to utilise machine learning methods to phenotype individuals at risk of KOA, by morphometric data.
**Method:** 3026 patients from the MOST database were recruited. Patients were followed up over 84 months. K-means and GMM clustering were used. The morphometric covariates used were leg length, knee height, height, BMI and HKA angle. The outcomes were incidence of KOA over 84 months, defined by progression to a KLG of ≥2, and WOMAC pain scores at baseline.

**Results:** 1454 left legs and 1397 right legs were eligible. The k-means model produced 2 distinct phenotypes and the GMM produced 5. In the k-means model, individuals with shorter, more-valgus legs had a greater risk of developing KOA (Left OR: 1.29, 95%CI 1.10-1.52, $p$=0.0196), (Right OR: 1.29, 95% CI: 1.06-1.45, $p$=0.0417) and greater pain scores (Left Δ: 0.56, 95%CI: 0.25-0.870, p<0.001), (Right Δ: 0.89, 95%CI: 0.57, 1.21, p<0.001).

In the GMM model, a cluster of shorter-legged, more-valgus individuals with higher BMI were identified, with the highest incidence of KOA (Left OR: 2.34, 95%CI: 1.56-3.52, p<0.001, Right OR: 2.71, 95%CI 1.89-3.88) and greater pain scores (Left mean: 3.42, 95%CI: 2.62-4.22, p<0.05), (Right mean: 3.82, 95% CI: 2.93-4.67, p<0.05).

**Conclusions:** Two clustering methods were used to phenotype individuals at risk of KOA. Across both methods, patients with shorter, more valgus legs and higher BMI were more likely to develop KOA within 84 months and had worse pain scores.

**270**

## Artificial intelligence in urology: transforming urolithiasis diagnosis

**Introduction:** Urolithiasis, prevalent at 12% in India, necessitates precise diagnosis. While non-contrast CT scans offer high accuracy, cost and radiation risks prevail. This project aims to develop an AI-driven X-ray KUB interpretation system for renal stone diagnosis, promising rapid and accurate assessments.

**Aim:** To create an AI program proficient in interpreting X-ray KUB for renal stone disease diagnosis, addressing resource constraints prevalent in Indian healthcare.

**Method:** Ethically sourced patient data underwent preprocessing and model development using TensorFlow's CNN architecture. Image resizing, normalization, and noise reduction were employed. Training, validation, and testing phases evaluated the model's diagnostic capabilities.

**Results:** In testing, the AI model achieved 86.66% accuracy in identifying right renal stones, 90% in left renal stones, 60% in bilateral stones, and 25% in negative cases. It correctly identified 65 out of 80 stone cases, yielding an overall accuracy of 0.7, sensitivity of 0.81, and specificity of 0.25.

**Conclusions:** Despite limitations compared to some existing models, this AI tool exhibits promise as a screening tool, particularly in resource-constrained settings. Larger datasets and improved computational resources may augment future models. The transformative potential of AI in urology remains evident, offering enhanced diagnostic avenues for renal stone disease.

**296**

## Machine learning for keloid scar management: a scoping systematic review

**Background:** The disfiguring and recurrent nature of keloid scars can lead to severe physical and psychological impacts on patients. Recent advancements in artificial intelligence (AI) present new opportunities to understand and manage the condition.

**Aim:** This scoping review examines the application of AI in diagnosing and managing keloid scars, highlighting key limitations and future research directions.

**Method:** Following the PRISMA-ScR (Preferred Reporting Items for Systematic Reviews and Meta-Analysis Extension for Scoping Reviews) guidelines, a systematic search was conducted in PubMed, Ovid MEDLINE, and Cochrane databases. Two reviewers selected and evaluated studies leveraging AI to investigate keloid scars. Data extracted included model architecture, prediction outcomes, accuracy and validity.

**Results:** Eight studies met the inclusion criteria. Five utilised machine learning (ML) to identify genes, metabolic pathways, and immune cells associated with keloid scarring, aiming to discover therapeutic targets. Most studies (three) reported area under curve (AUC) values exceeding 0.90, with all but two validating their findings using lab-based experimental data. The remaining three studies explored supervised and semi-supervised ML for image analysis to detect keloid scars, with two reporting algorithm accuracy above 90%. Data on model interpretability and transparency was limited in all studies.

**Conclusions:** Machine learning shows promise in keloid scar management. However, critical evaluation of algorithms and clinical validation are essential before implementation in clinical settings.

**317**

## Deep learning implementation to multispectral imaging and Laser Doppler Imaging to predict burn wound requirements for surgery

**Aim:** Burn injuries impair patients' quality of life and function. Accurate burn assessment remains a complex challenge. This study investigates the efficacy of an artificial intelligence-powered diagnostic tool which uses multispectral imaging (MSI) of the burn wound, in aiding medical teams in determining the appropriate course of conservative versus surgical management of burn wounds.

**Methods:** Thirty-one acute burn wounds, sustained by adult patients within 7 days of the injury, were assessed in the burn outpatient department of a national burn centre. Two devices were used as adjuncts: the AI-driven MSI wound assessment and Laser Doppler Imaging (NICE gold standard) device. Burn wounds located on the face, hands, feet, and genitals were excluded. The predictive outcomes generated by MSI and LDI were compared against the clinical outcome: conservative versus surgical management.

**Results:** The MSI AI-based device accurately predicted clinical outcomes in 58% of the cases, whereas LDI demonstrated a predictive accuracy of 90%. Concordance between MSI and LDI estimations was observed in 51.6% of cases.
**Conclusions:** This pilot study underscores the potential of AI technologies in augmenting clinical decision making processes in burn wound management. However, the binary classification approach employed by current AI models may not fully encapsulate the multifactorial nature of burn wounds. The moderate accuracy observed may reflect the inherent limitations of AI in capturing the complex pathophysiology of burns. Further refinement of AI models, in collaboration with clinical expertise, is imperative to develop innovative solutions that enhance the global delivery of burn care.

## Global surgery

**6**

### Safety of breast cancer surgery as a daycare procedure – outcome from a community hospital in a low middle income country (LMIC)

**Aim:** Evaluating the safety and practicality of outpatient breast cancer surgery in LMICs involves assessing postoperative complications, length of stay, and 30-day readmission rates following outpatient mastectomy.
**Method:** A retrospective observational cross-sectional study was conducted at the Cancer Foundation Hospital in Karachi, Pakistan from July 2019 to December 2020. It included 60 patients who underwent breast cancer surgery, with data recorded using electronic medical records. The study analysed patient demographics, disease characteristics, surgical procedures, and postoperative complications.
**Results:** The study included 58 female and 2 male patients with a mean age of 49 years and an average weight of 67 kg. 90% were diagnosed with Invasive Ductal Carcinoma (IDC), 36.7% had ER/PR-positive disease, and 23.3% had triple-negative breast cancer. 63.3% of the patients underwent a Modified Radical Mastectomy (MRM), and 76.7% received radiation therapy postoperatively. Reported complications included seroma in 8.3% of cases, aspiration in 1.667%, flap necrosis in 6.7%, wound infection in 5%, paraesthesia in 6.7%, and bleeding in 1.667%. No significant correlation was found between BMI, type of disease, and preoperative stage with the occurrence of complications.
**Conclusions:** Outpatient breast cancer surgeries, including mastectomies, have been shown to be safe and feasible in low- and middle-income countries (LMICs). Such ambulatory surgeries provide advantages such as fewer complications, efficient use of resources, and enhanced healthcare efficiency. The research underscores the importance of careful patient selection and points out the possibility of substantial cost reductions.

**20**

### National trends in incidence, mortality, and clinical outcomes of hospitalisations for thyrotoxicosis with and without thyroid storm in the United States, 2016–2020

**Aim:** Analysing trends in the incidence and outcomes of thyrotoxicosis can enhance our understanding of disparities in access and the quality of inpatient care. This study analysed recent trends in thyrotoxicosis hospitalizations with and without thyroid storm in the U.S. between 2016 and 2020.
**Methods:** We compared demographic characteristics using $\chi^2$ and linear regression analyses. We analysed trends in hospitalization rates, mortality, length of hospital stay, costs, and acute complications using Cuzick's tests.
**Results:** 33,430 hospitalizations for thyrotoxicosis were analysed. Hospitalization for thyrotoxicosis showed a downward trend from 7,444 (6.3 per 1000 hospitalizations for endocrine disorders) in 2016 to 5,424 (4.6 per 1000 hospitalizations for endocrine disorders) in 2020 ($P_{trend}$=0.002). Mortality rates showed an uptrend in hospitalizations without storm (10 [0.17%] in 2016 to 55 [1.30%] in 2020; $P_{trend}$<0.001). Mortality rates for thyroid storm increased from 10 (0.62%) to 50 (4.15%) in 2020; $P_{trend}$=0.051). There was uptrend in prolonged hospitalization in the total study cohort (11.9% [3,978] in 2016 to 14.6% [4,881] in 2020; $P_{trend}$=0.030). Thyroid storm was correlated with higher odds of any major adverse cardiovascular and cerebrovascular event (MACCE) (aOR: 1.05; 95% CI: 1.02-1.17; *P*=0.002), acute heart failure (aOR: 1.15; 95% CI: 1.03-1.78; P<0.001) and sudden cardiac death (aOR: 1.23; 95% CI: 1.04-2.17; *P*=0.041), and atrial fibrillation (aOR: 1.17; 95% CI: 1.05-2.06; P<0.001).
**Conclusions:** While overall hospitalizations for thyrotoxicosis decreased, mortality rates significantly increased, particularly in cases complicated by thyroid storm. There was a notable rise in healthcare costs, prolonged hospital stays, and the incidence of severe complications.

**99**

### Comparative meta-analysis of rescue surgical techniques for vascular emergencies in conflict zones: approaches and challenges in global surgery

**Aim:** This meta-analysis compares the effectiveness and challenges of rescue surgical techniques for vascular emergencies in conflict zones. With increasing global conflicts, it is crucial to evaluate different surgical approaches in resource-limited, high-risk environments, analysing success rates and complications.
**Method:** We analysed 32 studies from databases like PubMed, Scopus, and Cochrane, published between 2000 and 2023. Inclusion criteria focused on randomized and observational studies addressing emergency revascularization, direct vascular repairs, and temporary shunts. Data analysis was performed using RevMan 5.4 software with random-effects models. Heterogeneity was assessed with the $I^2$ test, and $p < 0.05$ was deemed significant.
**Results:** Among the 32 studies included, totalling 5,200 patients, direct vascular repair showed a 78% success rate (RR 1.42; 95% CI, 1.12–1.83; p = 0.02), while emergency revascularization had a 72% success rate (RR 1.31; 95% CI, 1.08–1.67; p = 0.04). The use of temporary shunts was effective in 60% of cases (RR

1.18; 95% CI, 0.94–1.42; p = 0.08), but was associated with higher complication rates, such as thrombosis and infections ($p < 0.001$). The $I^2$ test indicated moderate to high heterogeneity ($I^2 = 58\%$), reflecting variations in conflict settings and available resources.

**Conclusions:** The effectiveness of rescue techniques for vascular emergencies varies according to available resources and team experience. While direct repair has the highest success rate, the use of temporary shunts is crucial in extreme contexts, despite the higher risk of complications. Implementing training programs and developing standardized protocols are essential to improving surgical outcomes in conflict zones.

**105**

### Need for equitable access to minimally invasive surgery (MIS) in sub–Saharan Africa

**Aim:** Minimally invasive surgery (MIS) is increasingly being utilised and is fast replacing open surgeries in surgical practice across the world. However, its adoption is still very limited in low- and middle-income countries, despite great beneficial potential. This deficiency is largely attributed to inadequate training, poor health financing, amongst others. This paper examines the evolution of MIS in sub-Saharan Africa, addressing the challenges in access and providing recommendations for improvement.

**Method:** We conducted narrative review using published literature focused on access to MIS in sub-Saharan Africa and the extent to which this differs from what is obtainable in high-income countries.

**Results:** MIS is not being widely practised in sub-Saharan Africa majorly due to lack of sophisticated surgical equipment and dearth of experienced surgeons in the field. A range of obstacles has been identified as contributing factors, including insufficient government support and inadequate development and integration of MIS within healthcare systems. Potential solutions to address these challenges include enhanced training, improved health financing, stronger policy frameworks, advancements in digital health, and better data management. In addition, collaboration among stakeholders, including surgeons, training programs, and government policies, will promote the adoption of MIS.

**Conclusions:** Despite the challenges in adopting MIS in sub-Saharan Africa, there is significant potential for growth. However, it is crucial to raise awareness of existing inequities and implement strategies to ensure that MIS becomes more accessible.

**472**

### Sustainability in orthopaedic surgery: experience with the use of reprocessed biomaterials and devices across thirty-five hospitals in seven low and middle-income countries

**Aim:** The environmental impact of orthopaedic surgery is becoming an increasingly discussed topic. The popularity of single-use orthopaedic biomaterials, devices and equipment, especially in developed countries, continues to erode the principle of 'Reuse' that is being championed to promote environmental sustainability in orthopaedic surgery. We described the practice of using reprocessed implants, devices, and consumables (IDCs) in contemporary orthopaedic practice in seven low and middle-income countries to highlight the opportunities for greening operating theatres, healthcare cost savings and future developments towards ensuring safe practices.

**Method:** This was a multicentre cross-sectional study which assessed the practices of consultant orthopaedic surgeons, orthopaedic trainees, and career-grade orthopaedic specialists on the use of reprocessed orthopaedic IDCs using a web-based survey.

**Results:** Eighty-five responses were received from thirty-five hospitals across seven developing countries. 87% of respondents reported using reprocessed external fixators, 82% reprocessed tourniquets, and 47% reprocessed screws and orthopaedic wires and pins. Public non-profit hospitals used reprocessed implants more commonly than private fee-paying hospitals (87% vs 52%). 97% of respondents adopted reprocessed IDC because of cost savings. The most common reason for not using reprocessed IDC was a lack of awareness of the coordination and logistics of reprocessing. None of the centres had a clear policy on guidelines for the use of reprocessed IDC.

**Conclusions:** The study highlights the potential for meaningful change in orthopaedic practice to promote environmental sustainability in orthopaedic surgery, especially in developed countries. There is a need for further research to ensure safe practices in the use of reprocessed IDCs.

## Robotics and digital surgery

**30**

### Paediatric robotic colorectal surgery: a systematic review

**Introduction:** Robotic Assisted Laparoscopic Surgery (RALS) remains poorly utilised in paediatric surgery. RALS has been successfully compared to Conventional Laparoscopic Surgery (CLS) in paediatric urology and upper GI surgery. Little is known about the potential for RALS in paediatric colorectal surgery.

**Aims:** This study aims to evaluate paediatric colorectal RALS. The primary outcomes are the safety and efficacy of RALS compared to CLS. Secondary outcomes include cost-effectiveness and identification of key areas where RALS may be applied in the future.

**Methods:** A systematic review of the literature examined all studies describing paediatric colorectal RALS between 2013-2023. 366 records were identified. Following screening and retrieval, fourteen studies were included.

**Results:** Six nonrandomised comparative studies and 8 case series were examined including a total of 734 patients. 516 underwent RALS and 218 underwent CLS. Nine studies (64.2%), including five comparative studies, assessed RALS in Hirschsprung's disease. Five studies examined other colorectal pathology and procedures. The controlled studies of RALS in Hirschsprung's disease showed comparative outcomes to CLS, however operations took longer and were more expensive.
**Conclusion:** Paediatric colorectal RALS can be safe and effective. In Hirschsprung's disease, precise dissection can lead to comparable outcomes for patients compared with CLS. RALS has a faster learning curve compared to complex CLS. RALS remains expensive, and operations tend to take longer. Larger comparative studies are needed to assess where RALS can be more effective than CLS and in which aspects of paediatric colorectal surgery RALS is most useful.

**132**

### Early experience with robotic approaches to inflammatory bowel disease (IBD) surgery: a single institutional experience

**Aim:** Robotic approaches to colorectal resections are now becoming routine for colorectal cancer surgery but use in IBD is still not currently mainstream. Here, we compare our initial experience and 30-day surgical outcomes from three main surgical approaches for Inflammatory Bowel Disease (IBD), with specific focus on robotic outcomes.
**Method:** A single tertiary referral centre experience of all patients undergoing surgery for IBD between 2020–2023 was analysed. This retrospective review of all prospectively gathered surgical data utilised hospital database and prospectively recorded ERAS (Enhanced Recovery After Surgery) records detailing patient recovery and complications.
**Results:** 140 cases were analysed showing mean procedure time in minutes across robotic, laparoscopic and open approaches: ($p$=0.997) colectomies (268, 282, 265), panproctocolectomies (380, 425, 382), proctectomies (315, 259, 228) and small bowel resections (161, 165, 202). There was zero mortality across all groups. For robotic, laparoscopic and open, the major complications (Clavien-Dindo classification 3+) rates are 5.4%, 8% and 3.5% ($p$=0.686) and the readmission rates were 8.10%, 9.33% and 21.42% respectively. The mean postoperative stay in days ($p$=0.164): colectomies (6.70, 8.45, 10.33), panproctocolectomy (8.40, 8.92, 10.29), completion proctectomy (6.00, 5.00, 8.20) and ileocaecal/small bowel resection (6.42, 6.46, 6.80). The costs for patients undergoing robotic surgery were higher ($p$=0.11).
**Conclusions:** This early data demonstrates real-world experience of the introduction of robotic surgical techniques for colorectal resection. Robotic surgical outcomes demonstrate equivalence with the laparoscopic outcomes and are improved compared to open procedures in most metrics but are more expensive.

**361**

### Comparison of outcomes between stapled closure and sutured closure of pancreatic stump following robotic distal pancreatectomy

**Aim:** Postoperative pancreatic fistulas (POPFs) are associated with high patient mortality and morbidity. There are multiple approaches in managing pancreatic stumps following distal pancreatectomy. We compared the outcomes between sutured closure and stapled closure following robotic distal pancreatectomy and splenectomy, and the associated cost difference between the two techniques.
**Method:** We retrospectively analysed consecutive robotic distal pancreatectomy and splenectomy cases between October 2022 and January 2024 in a single United Kingdom tertiary hospital. Stapled closure was the initial standard approach. Sutured closure utilising 3/0 prolene was regularly performed from July 2023. The study cohort was divided into these two groups and outcomes were compared. Primary outcome was POPF rates and secondary outcomes included postoperative length of stay (LOS), 30-day readmission rates and postoperative complications graded by Clavien-Dindo classification (CDC). Equipment costs for the respective procedures were obtained from hospital procurement team.
**Results:** 36 patients were reviewed; 14 (38.9%) were stapled and 22 (61.1%) were sutured. Patient characteristics including mean age, gender and ASA grade were similar in both groups. 23/36 (63.9%) patients had confirmed malignancy on postoperative histopathology – similar distribution in both groups. 6/14 (42.9%) in the stapled group vs 4/22 (18.2%) in the sutured group developed POPF Grade B ($p$=0.14). LOS, 30-day readmission and CDC complication rates were similar in both groups. Median costs were £2236.40 and £1541.86 in the stapled and sutured groups respectively.
**Conclusions:** Sutured closure of pancreatic stump yielded similar outcomes to stapled closure following robotic distal pancreatectomy, associated with a significant cost saving in favour of sutured closure.

## Clinical research

**15**

### Anterior acetabular fracture repair via the modified Stoppa approach: Is there a soft tissue safe zone that can be identified intraoperatively to reduce the risk of iatrogenic injury to the obturator neurovascular bundle? (a dissection-based study)

**Aim:** The modified Stoppa approach is utilised in fixation of anterior acetabular fractures. Poor visibility of the posterior acetabulum makes it challenging to confirm the extra-articular placement of screws, posing a risk of injury to surrounding structures. Proposed "safe zones" in literature focus on osteological anatomy with a paucity of literature relating to soft tissue.

This study aimed to determine whether the linea terminalis can be used as an intraoperative landmark to aid when placing screws and reduce risk of injury to the obturator neurovascular bundle.
**Method:** 5 cadavers were dissected; a combination of qualitative and quantitative measurements were carried out to ascertain a "danger zone" for the obturator neurovasculature along the length of the linea terminalis.
**Results:** The obturator nerve crossed the linea terminalis within 25.00% to 37.04% of the total length of the linea terminalis. The obturator vessels lie within 12.00 mm to 26.00 mm from the linea terminalis at the site of the obturator canal. Presence of corona mortis variation was noted in all cadavers. No significant differences were found between left and right hemipelves or male and female cadavers.

**Conclusions:** This study highlights a potential "danger zone" within which the obturator nerves cross the linea terminalis. Obturator vessels lie within the osteological "safe zone" described in the literature and are at risk of iatrogenic injury. Presence of corona mortis variations in all 5 cadavers highlights the need for more research into defining a soft tissue "safe zone". The linea terminalis may be a useful intraoperative landmark to reduce risk of injury to obturator neurovasculature.

**60**

## Perianal abscess rupture and incidence of fistula formation after surgery – PARIS Study

**Aim:** Perianal abscesses are common anorectal conditions that can lead to significant complications, including perianal fistula formation. This study aims to assess the impact of abscess rupture status on the incidence of fistula formation following incision and drainage (I&D) procedures.

**Method:** This retrospective cohort study analysed data from 1,835 patients treated for perianal abscesses at a district general hospital between June 2019 and June 2023. Patients were categorized into two groups: those presenting with spontaneously ruptured abscesses and those with intact abscesses requiring I&D. The primary outcome was the incidence of fistula formation within one year post-procedure. Chi-square tests and multivariate logistic regression were used to identify predictors of fistula formation.

**Results:** The incidence of fistula formation was significantly higher in patients with spontaneously ruptured abscesses (54%) compared to those with intact abscesses (28.9%). Younger patients, particularly those under 50 years of age, were more likely to develop fistulas. Additionally, 67% of patients who developed fistulas had the external opening at the site of the original incision, indicating that the location of the incision may influence the type and complexity of the fistula.

**Conclusions:** Spontaneous rupture of perianal abscesses is associated with a higher risk of fistula formation. Early surgical intervention, careful incision placement, and consistent follow-up are recommended to reduce this risk and improve patient outcomes. The findings of the PARIS study highlight the importance of proactive management strategies in preventing long-term complications in patients with perianal abscesses.

**95**

## Evolving trends and future demands in ENT procedures: a 20-year analysis

A. Jangan, S. Minhas, E. Diakos, M. Simmons, Z. Mughal
Walsall Manor Hospital, Walsall, United Kingdom

**Aim:** This study aims to investigate the trends in otology, rhinology, and head and neck operations over the past two decades in England. These trends will allow for predictive modelling to forecast the demand over the coming decade in order to aid workforce and resource planning in ENT.

**Method:** Hospital episode statistics data were extracted between April 2000 and April 2023. A total of 121 otology, 114 rhinology, and 122 head and neck (H&N) procedure codes were included. Time series and linear regression were used to examine trends and produce a forecast model for volume of operations.

**Results:** A gradual upward trend in the volume of operations was observed across all three subspecialties. Rhinology showed the strongest linear trend ($R^2 = 0.844$), whereas H&N ($R^2 = 0.371$) and otology ($R^2 = 0.043$) displayed weaker trends. The COVID-19 pandemic significantly disrupted operating activity. To address the backlog and reach the pre-pandemic forecasted levels within the next five years, surgical capacity must increase by 6.4% in rhinology, 5.9% in otology, and 2.9% in H&N.

**Conclusions:** This analysis highlights a need for prioritization and expansion of the ENT workforce and resources to manage the current backlog and anticipated increase in demand over the next five years in England.

**177**

## The role of biomarkers in predicting recurrence in non-muscle invasive bladder cancer

**Aim:** This study aims to evaluate the prognostic value of urinary and tissue-based biomarkers in predicting recurrence in patients with non-muscle invasive bladder cancer (NMIBC). We seek to identify biomarkers that could enhance the current risk stratification models, improving individualized surveillance and treatment strategies.

**Method:** A prospective cohort study was conducted on 200 patients diagnosed with NMIBC between 2018 and 2022. Urine samples and tumour tissues were collected preoperatively and analysed for specific biomarkers, including urinary NMP22, FGFR3 mutations, and Ki-67 expression. Patients were followed up for a median of 24 months, with regular cystoscopies to detect recurrence. The correlation between biomarker expression and recurrence was analysed using Kaplan-Meier survival curves and Cox proportional hazards models.

**Results:** Of the 200 patients, 45% experienced recurrence during the follow-up period. High levels of NMP22 (>10 U/mL) and overexpression of Ki-67 (>20%) were significantly associated with higher recurrence rates ($p<0.01$). FGFR3 mutations were prevalent in 35% of cases but did not show a statistically significant correlation with recurrence ($p=0.23$). The combination of NMP22 and Ki-67 yielded the highest predictive accuracy, with a sensitivity of 82% and a specificity of 74%.

**Conclusions:** NMP22 and Ki-67 overexpression are valuable biomarkers for predicting recurrence in NMIBC. These markers, when used in conjunction with existing clinical models, could enhance risk stratification and guide more tailored surveillance protocols for patients at high risk of recurrence.

**206**

## Establishing a complex abdominal wall reconstruction multidisciplinary team for the North London region

**Aim:** Multidisciplinary teams (MDT) offer a coordinated and safe approach for planning complex surgical procedures. This project aims to establish a formal MDT for complex abdominal wall reconstruction (CAWR) surgery across the region. Previously, CAWR cases were discussed on an ad-hoc basis among consultants, leading to inconsistent documentation and fragmented care. This resulted in a backlog of patients awaiting surgery and compromised the quality of care.
**Method:** A waiting list was generated from consultants' existing caseloads. A standardised proforma was developed to guide the MDT and facilitate referrals. Meetings encompassing North London hospitals were held from February 2024 to present, with collaboration from plastic, general, and bariatric surgery. All discussions were documented in electronic patient records, and MDT recommendations were actioned.
**Results:** Over 10 MDT meetings, 34 cases were discussed. Of these, 13 cases were directly booked for surgery, with 5 surgeries completed. One case was removed due to a change in circumstances. Twenty cases were referred for pre-operative optimisation, including 15 for medical management of weight loss, 2 for conservative management of weight loss, and 3 for smoking cessation. A total of 14 cases were referred for pre-operative Botox therapy.
**Conclusions:** Through establishing this MDT, we achieved a streamlined and collaborative approach, improving the coordination and management of these complex cases. This enabled greater optimisation of patient care preoperatively and improvements in communication. This initiative highlights the value of a structured MDT in enhancing patient outcomes and ensuring a cohesive treatment pathway for complex surgical cases.

**439**

### Machine learning-based analysis of diagnostic strategies in acute appendicitis: optimising prediction of negative appendectomy rates and missed diagnoses

**Background:** Acute appendicitis diagnosis remains challenging due to the highly variable reliability of different diagnostic strategies, including blood tests and imaging. This variability contributes to significant negative appendectomies (NAR) rates and missed appendicitis (MIA), impacting patient care and resource utilization.
**Aim:** To employ machine learning (ML) algorithms to (1) assess the reliability of various diagnostic modalities in appendicitis and (2) identify factors associated with missed appendicitis and negative appendectomy rates.
**Method:** We retrospective analysed 387 patients who underwent appendectomies from 2018-2023. Patients were categorised into imaging-guided (Group A, *n*=326) and clinically-guided (Group B, *n*=61) groups. ML algorithms were applied to analyse the predictive value of imaging findings (US, CT), Alvarado score, and blood markers (WCC, neutrophil count, CRP) contributing to histopathological outcomes.
**Results:** The overall NAR was 7.4% (Group A: 6.2%, Group B: 16.4%). ML analysis revealed neutrophil count as the most reliable predictor (99% accuracy), followed by WCC (95.2%), CT findings (76.3%), CRP (30%), and Alvarado score (comparable to CRP). Ultrasound demonstrated the highest unreliability, missing 70.5% of appendicitis cases, compared to 2.8% for CT.
**Conclusions:** ML-driven analysis demonstrates that integrating CT imaging with blood markers, particularly neutrophil count and WCC, can significantly reduce NARs and MIA. This approach outperforms traditional clinical scoring and isolated use of CRP or ultrasound. Our findings suggest a refined diagnostic strategy for suspected appendicitis, emphasising the combination of differential blood count and targeted CT imaging to optimize diagnostic accuracy and patient outcomes.

## Early-stage innovation

**84**

### Continuously-calibrated diffuse reflectance spectroscopy for improved accuracy on real-time tissue classification

**Aim:** Accurate cancer margin assessment during surgery is crucial to ensure complete excision of malignant tissue. Traditional methods like histology are slow and labour-intensive. Diffuse reflectance spectroscopy (DRS) offers real-time assessment with high diagnostic accuracy, but systemic errors such as light source fluctuations and fibre bending losses hinder its performance. This study introduces a novel 'continuous calibration' technique to address these limitations and improve DRS reliability.
**Method:** Experiments were conducted using beef muscle, fat, and a 3D-printed cervical phantom. Systemic errors were validated, and data was collected and calibrated conventionally and continuously. The continuous calibration method captured reference spectra concurrently with target measurement spectra, providing real-time error correction. Logistic regression models were used to classify tissue types, and leave-one-out cross-validation was applied to evaluate accuracy.
**Results:** The continuous calibration technique significantly improved the stability and accuracy of light intensity measurements compared to conventional calibration. Continuously calibrated spectra exhibited reduced variability and more consistent mean intensity values. Diagnostic accuracy was significantly higher, with improvements observed in both beef samples ($p<0.0001$) and the cervical phantom ($p=0.0399$), attributed to enhanced real-time correction of systemic errors.
**Conclusions:** Continuous calibration presents a substantial improvement over conventional methods, offering greater measurement stability and diagnostic accuracy in DRS. This revolutionary technique has the potential to transform real-time tissue classification and surgical margin assessment. Future research should focus on validating this method in human tissues and exploring its application in surgical oncology. In vivo studies will be essential for fully assessing its impact and optimising integration into clinical practice.

**310**

### Photobiomodulation laser used in burn wound healing

**Aim:** Evaluate the efficacy of photobiomodulation (PBM) using K-Laser Blue in reducing the microbial load in burn wounds.
**Method:** Burn wounds were assessed in the outpatient department, during dressing change. A wound swab was performed before and after the photobiomodulation treatment. The pre- and post-laser microbial colony were counted on both swabs, after 24h of incubation.
**Results:** 43 pairs of swabs were evaluated and demonstrated a 78% reduction in microbial colony counts in the treated burn wounds compared to the baseline measurements taken before treatment.

**Conclusions:** Photobiomodulation with K-Laser Blue effectively reduces microbial load in burn wounds, as evidenced by a significant decrease in colony counts. This treatment could be a valuable adjunct in managing burn wounds by reducing infection risks and promoting better healing outcomes. Further research is needed to confirm these findings in burn wounds and wounds from different mechanisms of injury.

**323**

### Virtual arthroplasty follow-up (VARF)

**Aim:** Virtual Fracture Clinic is well established in orthopaedic surgery, but virtual arthroplasty follow-up is a novel concept. A successful pilot of virtual arthroplasty follow-up (VARF) was initiated in 2020 to review patients after Total Hip Arthroplasty (THR). The programme was designed to improve patient experience while reducing costs to the trust, the patient and the planet.
**Method:** A prospective study was conducted in a district general hospital for THR patients in 2022 + 2023. Patients were assessed based on their Oxford Hip Score (OHS), University of California, Los Angeles (UCLA) activity score, in addition to an x-ray at a health centre or hospital convenient to them. A patient satisfaction survey was undertaken via phone call. The environmental impact was estimated using CO2 emissions for driving and outpatient clinics taken from relevant literature.
**Results:** 319 THA patients were included. Overall, 73% had 'Good' or 'Excellent' OHS and 90% were successfully enrolled into the VARF pathway. 86% of patients were satisfied with the service and 63% very satisfied. Considering travel and clinic space, approximately 19 tonnes of CO2 was saved. Each patient saved £9.09 from travel and parking fees, and the Trust saved £51,736 from reduced face-to-face clinic time.
**Conclusions:** VARF has demonstrated an appropriate way to follow up THR patients while maintaining patient satisfaction, improving sustainability, and saving both the patient and trust expenses. After successfully implementing the programme for the THR patient, future work will focus on total knee replacement (TKR) patients to establish a similar strategy.

**360**

### A novel 3D printed fibula model for fracture fixation research

**Aim:** To create a novel 3D printed distal fibula model, precisely tuned to human anatomical and mechanical properties, to better emulate real-world conditions in a cost-effective manner.
**Method:** Drawing from existing literature, we determined the screw pull-out force of cadaveric fibula samples, focusing on both the diaphysis and distal fibula. We then tuned 3D printed samples of cortical and trabecular bone to mimic these properties, considering cortical thickness, trabecular infill density, and printer layer height. Subsequently, 3.5mm screws were inserted into 2.5mm pilot holes, and screw pull-out strength measurements were conducted using an Instron single-axis tensile tester.
**Results:** Literature revealed target parameters of 430N pull-out force for the distal fibula, and 800-1200N for the diaphysis. Optimisation based on these parameters gave a cortex with 3.25-3.5mm solid infill thickness proximally, a 32% gyroid infill density for the trabecular component, and a 0.2mm layer height. This configuration achieved screw pull-out forces within 10% of the cadaveric target.
**Conclusions:** Our study introduces a novel method for evaluating the biomechanics of fracture fixation, offering a realistic and customisable platform for simulating different fracture patterns and assessing fixation technique performance. By replicating fibular anatomical complexity, this model holds promise for enhancing fixation strategy evaluation and optimisation at reduced cost, and shorter lead time, than with commercially available synthetic bone.

**363**

### Biomechanical analysis of fibula fracture fixation: impact of screw configuration on torsional stiffness using a novel 3D printed model

**Aim:** To determine the most torsionally stiff neutralisation plate screw configuration among three common options, using a novel 3D printed fibula model.
**Method:** The novel 3D printed fibula was calibrated to replicate pull-out forces similar to cadaveric distal fibulae. Three screw configurations, two using a 7-hole 1/3 tubular plate and one using a 5-hole plate, were investigated: 123-567, 1-3-5-7, and 12-45. Four samples were prepared for each. Using a servohydraulic dual-axis Instron machine, the samples underwent loading with forces equivalent to walking (255N compression and 2Nm torsion at 0.02Hz). Optical trackers measured rotational amplitude to determine torsional stiffness.
**Results:** The 123-567 configuration yielded a mean rotational amplitude of 9.9° (range 9.6-10.5°, SD 0.5°), with a 1-3-5-7 configuration similar (mean 9.8°, range 8.0-11.6°, SD 1.8°). Conversely, the 12-45 configuration led to a small (16%) reduction in rotational amplitude (mean 8.4°, range 8.0-8.7°, SD 0.3°, $p$=0.014).
**Conclusions:** Using fewer screws led to minimal differences in rotational stiffness. Counterintuitively, the 5-hole plate with a 12-45 configuration was found to be the stiffest. Both 7-hole configurations demonstrated wider ranges, possibly influenced by the location of the tightest screw. Specifically, if the inner screw (in hole 3 or 5) was the tightest, it resulted in a stiffer construct compared to when the outer was tightest. Our study showcased the use of novel 3D printed fibulae in fracture fixation analysis, potentially refining surgical techniques by optimising screw placement. Use of fewer screws did not compromise torsional stiffness and may even have a positive effect due to less variable constructs.

## QI and audit

**219**

### The orthopaedic grab bag initiative: enhancing on-call efficiency in a major trauma centre (MTC)

**Aim:** MTC's deal with a high volume of polytrauma patients that require multiple specialty input. Subsequently, there are many patients with orthopaedic pathologies on non-orthopaedic wards with inconsistent supply of orthopaedic equipment. The on-call orthopaedic team spend significant time gathering equipment required for review and treatment of outlier patients.

We aim to assess whether a 'Grab Bag' of vital equipment (example: skin traction, joint aspiration equipment, splints, casting material) improved the efficiency of reviewing and treating outlier orthopaedic patients.

**Method:** A scoping review identified the time spent by on-call team gathering equipment to treat outlier patients. This also identified the equipment available on outlier wards, and the wards with poorest supply.

Subsequently, time to gather equipment for various procedures at various locations was compared with and without the use of the grab bag.

**Results:** An average of 45 minutes per 12-hour shift was spent locating equipment before the introduction of the grab bag.

The use of the grab bag improved the modal time for equipment gathering per procedure from 5-10 minutes (maximum 20-30 minutes) to 0-5 minutes (maximum 5-10 minutes).

The improvements were most notable when performing a joint aspiration (10 - 20 minutes → 5 - 10 minutes) and cast removal/reapplication (10-20 minutes → 0-5 minutes). All users surveyed reported that the grab bag enhanced their efficiency.

**Conclusions:** The implementation of the orthopaedic grab bag improved efficiency in gathering necessary equipment during on-calls, leading to faster patient review and treatment. These findings support the introduction of grab bags in other departments with substantial outlier patient populations.

**250**

## Innovative 3D printed simulation for peritonsillar abscess aspiration: a quality improvement project

**Aim:** Peritonsillar abscess (PTA) is the most common presenting deep neck space infection requiring prompt surgical drainage, a task often carried out by junior trainees in the otolaryngology department. Efficient training is important in ensuring quality aspiration technique, and medical simulation models have been proven to enhance clinical competence. Various simulation models have been proposed for use in teaching PTA aspiration techniques; however, they are limited in their accessibility, reproducibility, fidelity and cost. To address this deficit, a 3D printed simulation model of a peritonsillar abscess was introduced as a quality improvement project to advance current teaching methods of PTA aspiration techniques.

**Method:** Initial quantitative and qualitative feedback were collected from 12 trainees taught without the 3D printed simulation model. Based on these results, the peritonsillar abscess model was created using 3D printing techniques. Further feedback, adapted from the Michigan Standard Simulation Experience Scale (MiSSES), was collected from 27 trainees who were taught using the new model. This evaluated self-efficacy, fidelity, educational value, and teaching quality of the model on a five-point Likert scale.

**Results:** The new 3D printed model achieved statistically significant higher ratings on trainees' perception of their self-efficacy, the teaching quality and the overall training experience. Furthermore, 96% of trainees agreed the simulator can be used for training with slight or no further improvement. The model also achieved a high-fidelity rating (4.3 out of 5 average).

**Conclusions:** The development of our 3D printed peritonsillar abscess model introduces an innovative, high-fidelity and reproducible option to enhance the clinician's skills and confidence.

**252**

## Audit evaluating the safety and efficacy of transurethral laser ablation (TULA) and biopsy for recurrent bladder tumours and red patches

**Aim:** TULA and biopsy under local anaesthetic is a minimally invasive procedure used to manage recurrent non-muscle invasive bladder cancer (NMIBC) and biopsy suspicious red patches. Its precision and reduced anaesthetic risks make it particularly suitable for medically frail patients. This study evaluates its efficacy, safety, tolerance, tumour recurrence and tumour upgrading.

**Method:** A prospective audit was conducted in a tertiary hospital over 11 months (November 2023–September 2024). Data was collected on patient demographics, pain scores, complications, readmissions, cost analysis, and recurrence rates at three and six months.

**Results:** 62 patients underwent TULA, 77% were male, and the average age was 80. The average visual analogue pain score was 3 out of 10. Biopsies were taken in 57% ($n$=35) of cases, with 51% ($n$=18) of tumours identified as low-grade transitional cell carcinoma. Red patch biopsies were performed in 7 out of 9 patients. Complications were minimal, with 11% readmission due to haematuria. Tumour recurrence rate was 44% at three months and 15% at six months, with no reported cases of tumour upgrading. The average cost of TULA ranged from £250 to £300, significantly lower than the cost of £1,267 for TURBT.

**Conclusions:** TULA is a safe, well-tolerated, and cost-effective procedure for managing recurrent NMIBC in frail patients with minimal complications. It also facilitates effective biopsies of suspicious red patches to exclude carcinoma in situ, reducing the burden on the waiting list and preventing unnecessary anaesthesia. Overall, it has a low to moderate recurrence rate; however, larger studies are needed to validate these findings.

**364**

## Sustainability in breast surgery: an eco-audit of the breast implant pathway

Z. Ahmed[1], A. Zargaran[1,2], D. Marudamuthu[1], D. Zargaran[1,2], S. Sousi[1], J. Davies[1], S. Hamilton[2], A. Mosahebi[1,2]
[1]University College London, London, United Kingdom
[2]Royal Free NHS Foundation Trust, London, United Kingdom

**Aim:** Breast implants are the most common form of breast reconstruction performed globally. However, despite the vast number performed annually, the environmental impact of the pathway has yet to be evaluated.

**Method:** A retrospective analysis of 34 immediate breast implant reconstructions at a single reconstructive centre was performed. Process mapping and life-cycle analyses were performed for equipment, staff, patients and land. Carbon dioxide equivalent was used to estimate the relative contributions of the initial consultation, pre-operative, intra-operative and immediate post-operative periods.

**Results:** This study estimated the carbon footprint of a patient undergoing immediate reconstructive breast implant surgery to be approximately 48.80 kgCO2eq. The majority of emissions came from the intraoperative period (28.58kgCO2eq., 58.57%). Production and transport of surgical and anaesthetic equipment had the highest overall contribution to the carbon footprint (25.46kgCOZeq., 52.17% overall). Patient travel was the second greatest contributor, closely followed by anaesthesia (18.28 and 16.60% respectively).

**Conclusions:** This is the first study to estimate the carbon footprint of the breast implant pathway. Strategies to mitigate the impact of carbon emissions included the reuse of equipment. The impact of revisional procedures was not included and should be factored into future estimates of carbon footprint calculations.

**375**

### Modifications in surgical technique improve acute pain outcomes after conventional thoracotomy

**Aim:** Postsurgical pain remains a significant burden on patients undergoing thoracic surgery. We sought to evaluate the impact of introducing two stepwise modifications to conventional open thoracotomy on in-hospital outcomes.

**Method:** Patients undergoing first ipsilateral thoracotomy for pulmonary or mediastinal procedure from September 2023 to July 2024 were included. Three groups were identified: [1] conventional, [2] intercostal nerve sparing (ICNS), and [3] ICNS with cryoanalgesia (using the AtriCure CryoSphere device). Data was collected retrospectively from electronic and physical case records.

**Results:** 166 patients were included; 47.9% (*n*=80) male, age 66±13 years. There were 119 patients (71%) in the conventional group, 27 (16%) with ICNS, and 20 (12%) having ICNS+Cryoanalgesia.

Patients required less days of physiotherapy ($p$=0.042) during admission, across both in the ICNS (3.30 ± 1.99) and ICNS +Cryoanalgesia groups (1.86±1.24) vs. the conventional group (5.14 ± 7.02). Postoperative in-hospital morphine use showed a trend for reduced consumption but did not meet statistical significance. 25 patients (21%) in the conventional group suffered pulmonary complications, compared to 1 (4%) in ICNS and none after ICNS-Cryoanalgesia.

There was a significant reduction ($p$=0.003) in postoperative hospital stay in both ICNS (4.4±2.7 days) and ICNS-Cryoanalgesia groups (3.2±2.2), compared to conventional thoracotomy (7.1± 6.3).

**Conclusions:** Modifications in conventional surgical techniques can achieve in-hospital outcomes similar to those reported for minimally invasive approaches in large national trials. Both frugal and resource-intensive innovations seem to have value in this regard, with likely summative benefits. Prospective well-designed studies are required to validate these findings.

**464**

### Alveolar bone graft planning: ensuring children with cleft palate receive a timely specialist first-clinic for operative and dental extraction planning (KPI 11).

**Aim:** To improve the number and percentage of children in a designated birth year within the Salisbury "Spires" catchment area receiving their Alveolar Bone Graft (ABG) first joint specialist appointment between their 7th and 9th birthday. This is important for operative planning, so as not to offer ABG too early to impact facial growth, but to be before the permanent canines have erupted.

**Method:** (1) Search the Cleft Registry and Audit NEtwork (CRANE) database for all LAHSAL coded children born within the region between 2013 and 2015 (turning age 9 in 2022 to 2024, respectively). Sort into year of birth. (2) Confirm whether and when the first ABG clinic appointment occurred. (3) Highlight children needing their ABG appointment (prospective booking) and assign urgency by proximity to breaching (turning 9). (4) Create and share a 'Parent/Guardian Timeline' to support this.

**Results:** 2013 births showed of the 26 children, 14 breached the 9th birthday target and 3 were excluded (e.g., moved region), resulting in only 9/23 meeting the KPI (39%). The latest age was over 10 years old. 2014 births had 20 children, with 7 breaching and 2 excluded, resulting in 11/18 meeting the KPI (61%). The 2015 births had 24 children, 1 breached and 5 excluded, resulting in 15/16 children meeting the KPI (94%). The only child breaching in the 2015 cohort missed by 1.5 months only.

**Conclusions:** >50% Improvement in percentage of children receiving their ABG first clinic before their 9th birthday, by strengthening prospective booking practice using a birth-year LAHSAL spreadsheet.

## Systematic review and meta-analyses

**2**

### Performance enhancement using augmented reality during laparoscopic cholecystectomy: a systematic review and meta analysis

F. Cainelli, Z. Sahraoui, A. Achar, H. Sindhu
St George's University of London, London, United Kingdom

**Aim:** Use of augmented reality (AR) is a promising field in surgery regarding the enhancement of surgical precision and training. A comprehensive analysis of its efficiency in laparoscopic cholecystectomy remains to be evaluated. This systematic review and meta-analysis aims to appraise the pooled surgical precision outcomes of AR-augmented laparoscopic surgery.

**Method:** Searches across MEDLINE, Scopus, Google Scholar, and Cochrane Library were conducted for clinical trials exploring the role of AR in laparoscopic cholecystectomy between 2019 and 2023. The references of pertinent review articles were searched to identify additional clinical trials. A random-effects model was used to pool mean differences between studies with their 95% Confidence Interval (95% CI).

**Results:** This systematic review and meta-analysis included five RCTs with 198 surgeons. Compared to control techniques, AR-based laparoscopic cholecystectomy significantly improved the mean Global OSATS score (MD 2.07, 95% CI 0.34 to 3.80) and the mean Global GOALS score (MD 1.53, 95% CI 0.48 to 2.57). Additionally, the mean task-specific OSATS (MD 7.71, 95% CI 3.39 to 12.02) and mean task-specific GOALS scores (MD 1.04, 95% CI 0.25 to 1.83) also showed significant improvement with AR-augmentation.
**Conclusions:** AR augmentation during laparoscopic cholecystectomy can improve the efficiency of surgeons and ensure safety by fostering surgical precision. However, the small sample size for our pooled analysis necessitates future trials with robust designs and large sample size to develop a comprehensive understanding of the application of AR in surgical training and education.

**67**

## A systematic review of randomised control trials on patient-reported outcome measures in rhinoplasty

**Aim:** This systematic review explores patient-reported outcome measurements (PROMs) in rhinoplasty studies, aiming to guide future research and contribute to the development of optimised, validated PROMs for rhinoplasty.
**Method:** A PRISMA-compliant systematic review was conducted using bespoke search strategies applied to MEDLINE, Embase, Google Scholar, CENTRAL, Science Citation Index, and PubMed in February 2024. A protocol was developed and registered a priori (CRD42024513514). Risk of bias was assessed using Cochrane's Risk of Bias Tool and Cochrane ROBINS-I tool, for randomised controlled trials and observational studies, respectively. GRADE was used to assess the methodological quality of the studies.
**Results:** Twelve randomised controlled trials from 2006 to 2024 were included, with most studies originating from Iran. Risk of bias was moderate in nine studies, and significant heterogeneity was found in meta-analysed patient satisfaction scores ($I^2$ = 73%). Ten different PROMs were identified, with FACE-Q being the highest quality. The review was deemed the most methodologically robust according to AMSTAR-2 criteria.
**Conclusions:** The review found ten distinct PROMs across twelve studies, highlighting the need for a standardised, validated PROM tailored for rhinoplasty. Such a tool would enhance comparisons, improve understanding of quality of life, and identify areas for improvement, though care is needed due to the significant heterogeneity among studies.

**439**

## A systematic review of the efficacy and safety of autologous vs homologous cartilage grafts for dorsal augmentation rhinoplasty

**Aim:** Various graft materials, including septal, auricular, and costal cartilage, as well as alloplastic implants, have been utilized for dorsal augmentation rhinoplasty. Autologous costal cartilage (ACC) is frequently used due to its availability and strength, despite potential complications such as hypertrophic chest scars and pneumothorax. Irradiated homologous costal cartilage (IHCC) has emerged as an alternative to mitigate these risks. We conducted a high-quality systematic review to evaluate outcomes for ACC versus IHCC for dorsal augmentation.
**Methods:** The study was registered a priori on PROSPERO. MEDLINE, EMBASE, Google Scholar, and the Cochrane Central Register of Controlled Trials databases were searched up to October 2023. The main outcomes of interest were warping, resorption, revision rates, infection rates, and displacement.
**Results:** Out of 1527 articles identified, 43 studies with 2774 participants were included in the overall analysis. For both ACC and IHCC, warping was seen in 101/921 (11%) patients and 25/1252 (2%) patients respectively. 2% (16/760) patients in the ACC groups experienced resorption of graft compared to 6% (977/1235) in the IHCC group. 107/1492 (7%) patients had to undergo a revision surgery for various reasons in the ACC group compared to 49/1233 (4%) patients in the IHCC group. Upon completing the risk of bias assessment, the majority of the papers showed a serious risk of bias.
**Conclusions:** Robust evidence for superiority of one graft type over the other for dorsal augmentation rhinoplasty is lacking. Prospective, level I data, incorporating standardized clinical and patient-reported outcomes is urgently needed, to optimize informed consent.

**456**

## 2D simulators in surgical education: a systematic review, meta-analysis, appraisal of available apps, and the proposal of SET-2D framework

**Aim:** Technological advancements have enabled the development of mobile and web applications for surgical education. This review evaluates their effectiveness, available apps, and proposes a standardized assessment method.
**Method:** Registered on PROSPERO (CRD42024563275) and following PRISMA guidelines, this review involved: (1) Searching Medline, Embase, and PsycINFO (up to May 2024) for comparative studies on trainees, assessing scores, validity, acceptability, and usability; a random-effects meta-analysis was conducted with GRADE for quality assessment and Cochrane ROB2/ROBINS-I tools to assess bias risk. (2) Searching app stores for simulators, evaluated by price, rating, procedures, and assessment features. (3) Developing a tool to assess effectiveness with two students grading simulators.

**Results:** The search included 11 studies (5,761 trainees), all of which were high quality and low bias. Meta-analysis showed that 2D simulations significantly improved scores over traditional methods (MD=12.38; 95% CI: 1.84 to 22.91; $p$=0.02) but varied in validity and acceptability assessments; none explored usability. Nine apps were identified; 2 were paid, 1 had multiple languages, and the average rating was 4.55/5. Key themes were basic surgical skills (4/9) and general surgery (3/9), averaging 49 procedures. Briefing was present in 7 apps, debriefing in 6, and assessments in 7 (4 MCQs, 3 task-based). The SET-2D tool showed perfect concordance (Kappa=1.0).

**Conclusions:** The Technology Acceptance Model highlights ease of use, usability, and intention to use as key for adopting technologies like 2D simulators. Despite their effectiveness, lack of reporting on these factors' limits understanding of their use. A standardized framework, like the SET-2D tool, is needed to fill this gap.

**468**

### Oncologic and cosmetic outcomes of oncoplastic breast-conserving surgery after neoadjuvant systemic therapy: systematic review and meta-analysis

**Aim:** Oncoplastic breast-conserving surgery (OBCS) following neoadjuvant systemic therapy (NAST) for locally advanced or poorer prognosis tumours is under-recognised. This systematic review investigates outcomes (oncologic, surgical, cosmetic) of OBCS versus standard breast-conserving surgery (SBCS) and mastectomy.

**Method:** Electronic databases (Ovid, Cochrane, etc.) were searched up to 06/11/2022, updated search 24/08/2024. English language peer-reviewed RCTs or observational/cohort studies with ≥18-year-old women treated for breast cancer with NAST comparing OBCS to SBCS and/or mastectomy were included. A PRISMA guidelines-compliant review included two reviewers independently extracting data and assessing risk-of-bias (Newcastle-Ottowa Scale) utilising the system. A random-effects model was used. Measured oncologic outcomes included re-excision rate and overall survival.

**Results:** Of 5,271 articles, 32 underwent full-text assessment and eleven met the inclusion criteria. Eleven cohort studies included OBCS ($n$=912), SBCS ($n$=1,122) and mastectomy ($n$=2,560) after NAST.

Pre-NACT mean tumour size was 44.4(31.3-53)mm in the OBCS, 40(26.5-52.4)mm in the SBCS (SMD=0.40[0.08;0.71], $p$=0.01), and 42(40.5-43)mm in the mastectomy group (SMD=0.19[-0.02;0.41],$p$=0.08). The mean re-excision rate was 2.9%(0-11.1%) in the OBCS and 6.1%(0-18.5%) in the SBCS patients (OR=0.35[0.15;0.80],$p$=0.01).

Other oncologic outcomes were not statistically different between the groups. Limited data was available regarding surgical complications (OBCS2%-14% vs SBCS1%-9%). Cosmetic outcomes and patient-reported outcome measures were marginally in favour of OBCS (50-66% vs SBCS 37.6-55% very satisfied).

**Conclusions:** OBCS appears oncologically safe and a potential alternative following partial/poor tumour response to NAST. Studies comparing well-matched OBCS with SBCS and mastectomy patients after NAST may be useful.

## Best of the Best

**22**

### Leveraging artificial intelligence (AI) for automated polyp characterisation in endoscopy

**Aim:** Our aim is to employ ResNet architectures in developing a deep learning model for the objective characterization of adenomatous and hyperplastic polyps during endoscopic screening, while evaluating the performance of various ResNet variants to determine the most suitable depth for our objective.

**Method:** We implemented a deep metric learning algorithm using supervised image learning for NICE NBI classification. Utilising CNN with ResNet connections, specifically ResNet-18, ResNet-34, and ResNet-152 as feature extraction backbones, each representing increasing depth. During validation, the model's performance on the validation set was monitored over 200 epochs to track improvements or degradation, applying early stopping after 200 epochs if no performance gains were observed, with the best epoch selected based on the lowest validation loss.

**Results:** In our study, ResNet-34 emerged as the top overall performer, with the highest accuracy of 68.5% alongside a specificity of 79%, although its sensitivity trailed behind at 59% compared to ResNet-152's impressive 90%. Conversely, ResNet-18 showcased commendable precision at 82%, yet its accuracy and sensitivity stood at 54% and 46%. Despite ResNet-152's lower specificity at 30%, it still achieved a notable accuracy of 66%.

**Conclusions:** This study contributes valuable insights into AI-driven solutions for polyp classification, pinpointing ResNet-34 as a promising model with the potential to enhance the accuracy of polyp classification during endoscopic screening, thereby bolstering CRC prevention strategies.

**300**

### Perioperative optimisation in low- and middle-income countries (LMICs): a systematic review and meta-analysis of enhanced recovery after surgery (ERAS)

**Aim:** Enhanced recovery after surgery (ERAS) protocols have largely been incorporated into practice in high-income settings due to proven improvement in perioperative outcomes. We aimed to review the implementation of ERAS protocols and other perioperative optimisation strategies in low- and middle-income countries (LMICs) and their impact on length of hospital stay (LOS).

**Method:** We searched MEDLINE, PubMed, Global Health (CABI), WHO Global Index Medicus, Index Medicus, and Latin American and Caribbean Health Sciences Literature (LILACS) for studies incorporating ERAS or other prehabilitation approaches in LMICs. We conducted a pooled analysis of LOS using a random-effects model to evaluate the impact of such programs. This systematic review was pre-registered on PROSPERO.

**Results:** We screened 1,205 studies and included 70 for a full-text review; six were eligible for inclusion and five for quantitative analysis, two of which were randomised controlled trials. ERAS was compared to routine practice in all included studies, while none implemented prehabilitation or other preoperative optimisation strategies. Pooled analysis of 290 patients showed reduced LOS in the ERAS group with a standardised mean difference of -2.18 (95% confidence interval (CI) = -4.13, -.0.05, $p$<0.01). The prediction interval was wide (95% CI = -7.85, 3.48) with substantial heterogeneity ($I^2 = 94\%$).

**Conclusions:** Perioperative optimisation is feasible in LMICs and appears to reduce LOS, despite high levels of between-study heterogeneity. There is a need for high-quality data on perioperative practice in LMICs and supplementary qualitative analysis to further understand barriers to perioperative optimisation implementation.

**172**

## Robotic versus thoracoscopic minimally invasive mitral valve surgery: a systematic review and meta-analysis of matched studies

**Aim:** Mitral regurgitation (MR) is the most prevalent form of valvular heart disease, impacting over 24 million people globally. Robotic and thoracoscopic minimally invasive mitral valve repair (MIMR) techniques have emerged as viable alternatives to traditional open-heart surgery. However, the comparative effectiveness and safety of these two approaches remain underexplored. This systematic review and meta-analysis, conducted according to PRISMA guidelines, aimed to compare robotic and thoracoscopic MIMR outcomes.

**Method:** A literature search was performed across PubMed, Scopus, and Embase databases to identify studies comparing these surgical techniques. Eligible studies included randomized controlled trials and cohort studies. Six propensity-score-matched studies and one retrospective cohort study, involving 11,823 patients were included, with 5,851 undergoing robotic mitral valve repair and 5,972 receiving thoracoscopic MIMR.

**Results:** No significant differences were found in perioperative mortality (RR 0.97, 95% CI [0.65–1.45]) or pump/clamp times. Robotic surgery was associated with longer operative times (Mean Difference: 33.01 minutes) and higher intraoperative transfusion rates (RR 1.53, 95% CI [1.07–2.18]), but a lower risk of atrial fibrillation (RR 0.89, 95% CI [0.83–0.95]).

**Conclusions:** Robotic and thoracoscopic MIMR show comparable mortality and overall safety profiles. However, robotic surgery may require longer operative times and increased transfusion needs, while reducing the risk of atrial fibrillation. Further high-quality, randomized studies are warranted to validate these findings.

**39**

## Approaching orthognathic advancement: a systematic review and meta-analysis comparing the stability of surgically-assisted and orthodontic maxillary protraction techniques

**Aim:** This systematic review and meta-analysis aimed to compare the stability of two techniques for maxillary protraction in orthognathic advancement surgery.

**Method:** Following Cochrane Handbook and PRISMA guidelines, searches were conducted across PubMed, Embase, Cochrane, and Web of Science. Peer-reviewed English studies on maxillary protraction stability and surgical complications were included. Data were extracted and analysed using IBM SPSS and Microsoft Excel. Bias was assessed with the Cochrane Risk of Bias Tool (RoB2), and the review was registered on PROSPERO (CRD42024509005).

**Results:** Eight studies with 316 patients were reviewed. Surgical-assisted maxillary protraction (SAMP) showed greater increases in arch width (3.5 mm ± 1.2 mm vs. 1.8 mm ± 0.7 mm), palatal width (2.6 mm ± 0.8 mm vs. 1.2 mm ± 0.4 mm), and upper anterior teeth inclination (4.2° ± 1.5° vs. 2.3° ± 0.9°) compared to orthodontic maxillary protraction (OMP). SAMP also led to a higher mean increase in the SNA angle (3.1° ± 1.1° vs. 1.7° ± 0.6°), with these changes being statistically significant ($p < 0.05$).

**Conclusions:** SAMP demonstrated superior outcomes in arch morphology, dental shifts, and skeletal changes compared to OMP. SAMP offers faster and more significant changes, while OMP provides stable, long-term results, especially for younger patients. The choice between SAMP and OMP should be tailored to patient needs, with consideration of age, goals, and potential risks. This study highlights the need for long-term research to further evaluate the efficacy and risks of these techniques in orthognathic surgery.

**339**

## Evaluation of volatile organic compounds as non-invasive biomarkers for early detection of hepatocellular carcinoma and cholangiocarcinoma: a systematic review

**Aim:** Hepatocellular carcinoma (HCC) and cholangiocarcinoma (CCA) are the most common types of primary liver cancer, often detected at late stages, leading to poor prognosis. Non-invasive biomarkers for early detection are crucial to improve survival. Volatile organic compounds (VOCs) emitted during cellular metabolism have emerged as potential biomarkers. This systematic review evaluates the evidence on VOCs for early detection of HCC and CCA, focusing on their sensitivity, specificity, and diagnostic performance.

**Method:** A systematic search of PubMed, Scopus, and Web of Science was conducted for studies published from January 2010 to June 2023 that investigated VOCs in human biological samples for detecting HCC or CCA. Studies were included if they reported on the sensitivity and specificity of VOCs as biomarkers. Data on study design, sample size, types of biological samples, VOCs identified, detection methods, and diagnostic accuracy were extracted. The Newcastle-Ottawa Scale (NOS) was used to assess study quality.

**Results:** Twenty-four studies met the inclusion criteria, with a combined sample size of 2,340 participants. Breath samples were most commonly analysed (18 studies), followed by blood (4) and urine (2). Common VOCs identified in HCC included ethanol, acetone, and hexanal; for CCA, octane, nonanal, and 2-propanol were predominant. Pooled sensitivity and specificity for VOC-based detection were 81% and 74%, respectively.

**Conclusions:** VOCs show promise as non-invasive biomarkers for early detection of HCC and CCA. However, variability in detection methods and external factors limit their current clinical application. Further research should aim to standardize methods and validate findings in diverse cohorts.

**Open Access** This is an open-access article distributed under the terms of the Creative Commons Attribution 4.0 International License, which permits unrestricted use, distribution, reproduction, and adaptation in any medium, provided the original work is properly attributed.


Subjects: Artificial Intelligence in Surgery

106 Addressing inequalities in AI-powered women’s health solutions in low- and middle-income countries

Amusat O. 1
Togunwa T. 2 3
Babatunde A. 2 3
Adeyeye O. 2
Abiona F. 2
Aborode A. 4
1 Luton and Dunstable University Hospital, Luton, United Kingdom
2 College of Medicine, University of Ibadan, Ibadan, Nigeria
3 College Research and Innovation Hub, University College Hospital, Ibadan, Nigeria
4 Department of Research and Development, Healthy Africans Platform, Ibadan, Nigeria

131 Optimising pancreas detection in organ retrieval photography with deep learning automation

Duraisaminathan Valli A. 1
Kourounis G. 2 3
Ahmed Elmahmudi A. 4
Thomson B. 4
Tingle S.J. 5 3
Mahendran B. 2 3
Thompson E. 2 3
Hunter J. 6
Ugail H. 4
Wilson C. 2 3
1 Newcastle University Medical School, Newcastle, United Kingdom
2 NIHR Blood and Transplant Research Unit, Newcastle and Cambridge University, Newcastle, United Kingdom
3 Institute of Transplantation, The Freeman Hospital, Newcastle, United Kingdom
4 Centre for Visual Computing and Intelligent Systems, Faculty of Engineering and Informatics, Bradford University, Bradford, United Kingdom
5 NIHR Blood and Transplant Research Unit, Newcastle University and Cambridge University, Newcastle, United Kingdom
6 Nuffield Department of Surgical Sciences, University of Oxford, Oxford, United Kingdom

142 Morphological phenotyping of knee osteoarthritis using machine learning methods

Tooke J. 1 2
Musbahi O. 1
Jones G. 1
1 Imperial College London, London, United Kingdom
2 University of Manchester, Manchester, United Kingdom

270 Artificial intelligence in urology: transforming urolithiasis diagnosis

Chandrashekhar S. 1
Bahrani P. 2
Roy A. 3
Shekhar T. 4
Khan N. 5
Shekhar T. 6
1 SRM Medical College Hospital and Research Centre, Chennai, India
2 Freshworks, Chennai, India
3 SRMIST, Chennai, India
4 MGM Medical College, Hospital and Research Centre, Navi Mumbai, India
5 Royal Sussex County Hospital, Brighton, United Kingdom
6 Northeastern University, Boston, USA

296 Machine learning for keloid scar management: a scoping systematic review

Ejaz H. 1
Aftab S.B. 2
Frew Q. 1
1 St Andrew's Centre for Plastic Surgery and Burns, Mid Essex Hospital Services NHS Trust, Chelmsford, United Kingdom
2 Department of General Surgery, Pakistan Institute of Medical Sciences, Islamabad, Pakistan

317 Deep learning implementation to multispectral imaging and Laser Doppler Imaging to predict burn wound requirements for surgery

Pérusseau-Lambert A.
Akram M.
Frew Q.
Barnes D.
St Andrew's Centre for Burns, Plastic and Reconstructive Surgery, Chelmsford, United Kingdom

Subjects: Global Surgery

6 Safety of breast cancer surgery as a daycare procedure – outcome from a community hospital in a low middle income country (LMIC)

Maha Q. A. 1
Talal M. 2
1 Nottingham University Hospital, Nottingham, United Kingdom
2 Aga Khan University Hospital, Karachi, Pakistan

20 National trends in incidence, mortality, and clinical outcomes of hospitalisations for thyrotoxicosis with and without thyroid storm in the United States, 2016–2020

Asogwa S. 1
Uwumiro F. 2
Asaju F. 3
1 London North West University Healthcare NHS Foundation, London, United Kingdom
2 University of Jos College of Health Sciences, Jos, Nigeria
3 Dorset County Hospital NHS Foundation Trust Williams Avenue, Dorchester, United Kingdom

99 Comparative meta-analysis of rescue surgical techniques for vascular emergencies in conflict zones: approaches and challenges in global surgery

Santos Samaritano Pereira M.
Íscaro Andrade T.
Góes Silva V. H.
Universidade Municipal de São Caetano do Sul, Itapetininga, Brazil

105 Need for equitable access to minimally invasive surgery (MIS) in sub–Saharan Africa

Amusat O. 1
Abdulrasheed H. 2
Omoniyi N. 3
Babatunde A. 3
1 Luton and Dunstable University Hospital, Luton, United Kingdom
2 University Hospitals Birmingham NHS Foundation Trust, Birmingham, United Kingdom
3 College of Medicine, University of Ibadan, Ibadan, Nigeria

472 Sustainability in orthopaedic surgery: experience with the use of reprocessed biomaterials and devices across thirty-five hospitals in seven low and middle-income countries

Oladeji E. 1 2
Olatide O. 2 3
Awi O. 2 4
Abdullateef R. 2 5
Ringim A. 2
Abdulquddus Ajibade A. 5
Zubair A. 2
Olayode O. 2
Okonkwo P. 2 1
Olaniyi A. 2
REBOOT Study Collaborative 2
1 St Richard's Hospital, Chichester, United Kingdom
2 Surgery Interest Group of Africa, Lagos, Nigeria
3 LAUTECH Teaching Hospital, Ogbomoso, Nigeria
4 University College Hospital, Ibadan, Nigeria
5 College of Medicine University of Ibadan, Ibadan, Nigeria

Subjects: Robotics and Digital Surgery

30 Paediatric robotic colorectal surgery: a systematic review

Balfour J. 1 2
Clarke M. 3
1 Royal Hospital for Children, Glasgow, United Kingdom
2 University of Edinburgh, Edinburgh, United Kingdom
3 Royal Aberdeen Children’s Hospital, Aberdeen, United Kingdom

132 Early experience with robotic approaches to inflammatory bowel disease (IBD) surgery: a single institutional experience

Jenny N.E.J.
Thattaruparambil V.
Konkoth A.
Brady R.
Kay R.
Randhawa N.
Newcastle upon Tyne NHS Hospitals Foundation Trust, Newcastle Upon Tyne, United Kingdom

361 Comparison of outcomes between stapled closure and sutured closure of pancreatic stump following robotic distal pancreatectomy

Ugur S.
Kho J.
Hamady Z.
Arshad A.
University Hospital Southampton NHS Foundation Trust, Southampton, United Kingdom

Subjects: Clinical Research

15 Anterior acetabular fracture repair via the modified Stoppa approach: Is there a soft tissue safe zone that can be identified intraoperatively to reduce the risk of iatrogenic injury to the obturator neurovascular bundle? (a dissection-based study)

Asif H.
University of Keele, Keele, United Kingdom. East Sussex NHS Trust, East Sussex, United Kingdom

60 Perianal abscess rupture and incidence of fistula formation after surgery – PARIS Study

Shaikh A. G.
Kingston Hospital, London, United Kingdom

95 Evolving trends and future demands in ENT procedures: a 20-year analysis

Jangan A.
Minhas S.
Diakos E.
Simmons M.
Mughal Z.
Walsall Manor Hospital, Walsall, United Kingdom

177 The role of biomarkers in predicting recurrence in non-muscle invasive bladder cancer

Shaker G.S.H.
Shaker M.
North Tees and Hartlepool NHS trust, Stockton-on-Tees, United Kingdom

206 Establishing a complex abdominal wall reconstruction multidisciplinary team for the North London region

Eaton D. 1
Awad L. 1
Ghali S. 1
Markakis H. 2
Hart C. 1
Al-Ajam Y. 1
1 Royal Free Hospital, London, United Kingdom
2 University College London Hospital, London, United Kingdom

439 Machine learning-based analysis of diagnostic strategies in acute appendicitis: optimising prediction of negative appendectomy rates and missed diagnoses

Hakeem A. 1 2
Viswanathan P. 1
Ali A. 1
1 University Hospital Ayr, Ayr, United Kingdom
2 Altnagelvin Hospital, Londonderry, United Kingdom

Subjects: Early-Stage Innovation

84 Continuously-calibrated diffuse reflectance spectroscopy for improved accuracy on real-time tissue classification

Yang H.
Imperial College London, London, United Kingdom

310 Photobiomodulation laser used in burn wound healing

Pérusseau-Lambert A.
Broome E.
Barnes D.
Frew Q.
St Andrew's Centre for Burns, Plastic and Reconstructive Surgery, Chelmsford, United Kingdom

323 Virtual arthroplasty follow-up (VARF)

Platt N.
Bolland B.
Musgrove Park Hospital, Taunton, United Kingdom

360 A novel 3D printed fibula model for fracture fixation research

Lovat B. 1
Marsden S. 2
Trompeter A. 3
van Arkel R. 1
1 Imperial College, London, United Kingdom
2 Mersey Deanery, Liverpool, United Kingdom
3 St George's University Hospital, London, United Kingdom

363 Biomechanical analysis of fibula fracture fixation: impact of screw configuration on torsional stiffness using a novel 3D printed model

Lovat B. 1
Marsden S. 2
Trompeter A. 3
van Arkel R. 1
1 Imperial College, London, United Kingdom
2 Mersey Deanery, Liverpool, United Kingdom
3 St George's University Hospital, London, United Kingdom

Subjects: QI and audit

219 The orthopaedic grab bag initiative: enhancing on-call efficiency in a major trauma centre (MTC)

Datta R.
Vusirikala A.
Li L.
St Mary’s Hospital - Imperial College Healthcare NHS trust, London, United Kingdom

250 Innovative 3D printed simulation for peritonsillar abscess aspiration: a quality improvement project

Umelo C. 1
Shetty P. 2
1 NHS, London, United Kingdom
2 NHS, Brighton, United Kingdom

252 Audit evaluating the safety and efficacy of transurethral laser ablation (TULA) and biopsy for recurrent bladder tumours and red patches

Madasu A. 1
Anwar A. 2
Panaretos G. 2
Basu J. 2
1 Calderdale and Huddersfield NHS Foundation Trust, Halifax, United Kingdom
2 Bradford Teaching Hospitals NHS Foundation Trust, Bradford, United Kingdom

364 Sustainability in breast surgery: an eco-audit of the breast implant pathway

Ahmed Z. 1
Zargaran A. 1 2
Marudamuthu D. 1
Zargaran D. 1 2
Sousi S. 1
Davies J. 1
Hamilton S. 2
Mosahebi A. 1 2
1 University College London, London, United Kingdom
2 Royal Free NHS Foundation Trust, London, United Kingdom

375 Modifications in surgical technique improve acute pain outcomes after conventional thoracotomy

Regmi B. 1
Clayton S. 2 3
Hassan L. 2
Caruana E.J. 2 4
1 Thoracic Surgery, Glenfield Hospital, Leicester, UK, Leicester, United Kingdom
2 Thoracic Surgery, Glenfield Hospital, Leicester, United Kingdom
3 University of La Laguna Medical School, Santa Cruz de Tenerife, Spain
4 Institute for Lung Health, NIHR Leicester Biomedical Research Centre, Dept of Respiratory Sciences, University of Leicester, Leicester, UK, Leicester, United Kingdom

464 Alveolar bone graft planning: ensuring children with cleft palate receive a timely specialist first-clinic for operative and dental extraction planning (KPI 11).

Honey J. 1
Fallico N. 2
Bussell M. 2
1 Swansea University Health Board, Swansea, United Kingdom
2 Salisbury District Hospital, Salisbury, United Kingdom

Subjects: Systematic Review and Meta-Analyses

2 Performance enhancement using augmented reality during laparoscopic cholecystectomy: a systematic review and meta analysis

Cainelli F.
Sahraoui Z.
Achar A.
Sindhu H.
St George's University of London, London, United Kingdom

67 A systematic review of randomised control trials on patient-reported outcome measures in rhinoplasty

Rupra R. 1
Daneshi K. 2
Naidoo S. 3
Chauhan S. 4
Imantalab D. 5
Alaa B. 6
Vyas K. 7
Khajuria A. 8 9
1 James Paget University Hospital, Great Yarmouth, United Kingdom
2 The University of Sheffield, Sheffield, United Kingdom
3 Stellenbosch University, Stellenbosch, South Africa
4 Kalam Experts, Visakhapatnam, India
5 Keele University, Keele, United Kingdom
6 Royal Berkshire Hospital NHS Foundation Trust, Reading, United Kingdom
7 Massachusetts General Hospital and Harvard Medical School, Boston, USA
8 Kellogg College, University of Oxford, Oxford, United Kingdom
9 Imperial College London, London, United Kingdom

439 A systematic review of the efficacy and safety of autologous vs homologous cartilage grafts for dorsal augmentation rhinoplasty

Liyanage D.
Queen Elizabeth Hospital Birmingham, Birmingham, United Kingdom

456 2D simulators in surgical education: a systematic review, meta-analysis, appraisal of available apps, and the proposal of SET-2D framework

Khademi Mansour H. R. 1
Tehranchi S. 2
Al-Hashemi S. 3
Zargaran A. 4 5
Zargaran D. 4 5
Mosahebi A. 4 5
1 King's College London, London, United Kingdom
2 Queen Mary University of London, London, United Kingdom
3 University College Dublin, Dublin, Ireland
4 University College London, London, United Kingdom
5 The Royal Free Hospital, London, United Kingdom

468 Oncologic and cosmetic outcomes of oncoplastic breast-conserving surgery after neoadjuvant systemic therapy: systematic review and meta-analysis

Baron D. 1
Ahmed G. 2
Agrawal A. 3
1 Oxford University Hospitals NHS Foundation Trust, Oxford, United Kingdom
2 NHS Frimley Health Foundation Trust, Frimley, United Kingdom
3 Cambridge University Hospitals NHS Foundation Trust, Cambridge, United Kingdom

Subjects: Best of the Best

22 Leveraging artificial intelligence (AI) for automated polyp characterisation in endoscopy

Hammad A. 1 2
Ali S. 3
1 University of York, York, United Kingdom
2 University of Hull, Hull, United Kingdom
3 University of Leeds, Leeds, United Kingdom

300 Perioperative optimisation in low- and middle-income countries (LMICs): a systematic review and meta-analysis of enhanced recovery after surgery (ERAS)

Riad A. 1
Barry A. 1
Knight S. 2
Arbaugh C. 3
Haque P. 4
Weiser T. 5
Harrison E. 2
1 Royal Infirmary of Edinburgh, Edinburgh, United Kingdom
2 Centre for Medical Informatics, University of Edinburgh, Edinburgh, United Kingdom
3 Department of Surgery, Stanford Health Care and Stanford University, California, USA
4 Department of General Surgery, Christian Medical College and Hospital, Punjab, India
5 Department of Surgery, Stanford Health Care and Stanford University, California, United Kingdom

172 Robotic versus thoracoscopic minimally invasive mitral valve surgery: a systematic review and meta-analysis of matched studies

De Jesus J. 1
Estrella J. 1
Jesse J. 2
Binny V. 3
SRP P. 4
Kabir Y. 5
Nallamotu S. 6
Guntupalli S.V. 7
yin Cai L. 8
Al-Tawil M. 9
1 Queen Alexandra Hospital, Portsmouth, United Kingdom
2 Universidad de Montemorelos, Nuevo Leon, Mexico
3 Gretchen Whitney High School, Cerritos, USA
4 Tbilisi State Medical University, Tbilisi, Georgia
5 Hywel Dda University Health Board, Carmarthen, United Kingdom
6 Kasturba Medical College, Manipal, India
7 Guntur Medical College, Guntur, India
8 Caribbean Medical University, Willemstad, Curaçao
9 Faculty of Medicine, Al-Quds University, East Jerusalem, Palestine

39 Approaching orthognathic advancement: a systematic review and meta-analysis comparing the stability of surgically-assisted and orthodontic maxillary protraction techniques

Yassa B.
Barts and the London School for Medicine and Dentistry, London, United Kingdom

339 Evaluation of volatile organic compounds as non-invasive biomarkers for early detection of hepatocellular carcinoma and cholangiocarcinoma: a systematic review

Karagiannidis G. 1
Morsi-Yerogiannis A. 2
1 Imperial College London, London, United Kingdom
2 Ippokrateio Hospital, Thessaloniki, Greece

